# Sarcopenia, a Neurogenic Syndrome?

**DOI:** 10.1155/2013/791679

**Published:** 2013-03-13

**Authors:** Ping Kwan

**Affiliations:** ST013a, Department of Rehabilitation Sciences, Hong Kong Polytechnic University, Hong Kong

## Abstract

Sarcopenia is an aging-associated condition, which is currently characterized by the loss of muscle mass and muscle strength. However, there is no consensus regarding its characterization hitherto. As the world older adult population is on the rise, the impact of sarcopenia becomes greater. Due to the lack of effective treatments, sarcopenia is still a persisting problem among the global older adults and should not be overlooked. As a result, it is vital to investigate deeper into the mechanism underlying the pathogenesis of sarcopenia in order to develop more effective therapeutic interventions and to inscribe a more uniform characterization. The etiology of sarcopenia is currently found to be multifactorial, and most of the pharmacological researches are focused on the muscular factors in aging. Although the complete mechanism underlying the development of sarcopenia is still waiting to be elucidated, we propose in this article that the primary trigger of sarcopenia may be neurogenic in origin based on the intimate relationship between the nervous and muscular system, namely, the motor neuron and its underlying muscle fibers. Both of them are affected by the cellular environment and their physiological activity.

## 1. Introduction

Sarcopenia (Greek: *sarx* means “flesh,” *penia* means “loss”) is an age-related geriatric syndrome first described on a meeting in 1988 by Dr. Rosenberg as a phenomenon whereby the age-related decline in lean body mass affects ambulation, mobility, energy intake, overall nutrient intake and status, independence, and breathing [1]. More recently, sarcopenia is characterized by the gradual loss of muscle mass, muscle strength, and muscle function/quality in aging [2, 3]. However, there are studies indicating that sarcopenia should be referred only as the age-related loss in muscle mass whilst the age-related loss in muscle strength should be isolated as a new condition called “dynapenia” based on the evidences indicating that the loss of muscle mass and strength are two distinct processes with different pathophysiology [4].

Hitherto, there is no consistent data among a variety of prevalence studies probably due to the difference in study sample, definition of sarcopenia, and the assessment tool used [3]. For example, for the population of over 80 years old, American study of New Mexico Elder Health Survey has found that there were >40% of women and 50% of men who were sarcopenic whilst the NHANES III database study reveals that 11% of women and 7% of men were sarcopenic. Also, the Italian study of InCHIANTI cohort reveals that 15% of women and 70% of men were sarcopenic [3]. In summary, the prevalence of this syndrome depends on age, sex, race [3, 5–7], morbidity [7, 8], nutrition [9], and physical activity [10, 11].

Some studies indicate that sarcopenia leads to functional limitations, impaired mobility, disability, falls, and fractures, which in turn lead to the loss of independence, frailty, and increased risk of mortality [11–13]. In contrast, some studies indicate that the parameter of muscle mass may not relate to the functional status and mortality [4]. No matter whether sarcopenia may affect both the quality and quantity of life of individuals or not, there are no effective treatment for sarcopenia and its related outcomes yet. Pharmacological interventions currently present are not effective and possess a considerable level of side effects. These include hormonal (e.g., growth hormones, testosterones, estrogens, tibolone) and nonhormonal (e.g., losartan, telmisartan) interventions [14]. Hitherto, only exercise training except endurance training, especially by power training [11, 14], and with adequate nutrition [9] seems to be the relatively effective intervention with the least side effects. Together with the inconformity of sarcopenia characterization among different studies, it is imperative to explore deeper into the mechanism or etiology of sarcopenia which in turn will provide further insight into the development of more effective therapy as well as a better understanding of the rationale that leads to a better characterization of sarcopenia.

In this article, we aimed to provide another perspective on sarcopenia and propose that sarcopenia may be neurogenic in nature despite its muscle-related features. In the following sections, we will discuss first the definition of sarcopenia, followed by commonly accepted etiology of sarcopenia, and then the impact and overview of the physiological factors that link up the nervous and muscular system.

## 2. Definition of Sarcopenia

Despite the characterization of sarcopenia varied among different studies [2–4, 15, 16], the major impact of sarcopenia is the age-related progressive decline in muscle mass. Under normal circumstances, when a healthy individual is trying to perform a particular physical activity, the relevant muscles on the corresponding part of the body will start to generate strength or force by neural action potential-induced contraction. The part of the body moves only when the overall strength produced overcomes the weight exert by the corresponding body part and the resistance forces induced by the activity. It is commonly assumed that if every muscle fiber of a single type has the same rate of force generation and produces the same force, then the muscle strength shall be positively correlated with the number of muscle fibers present in the muscle. Thus, the loss of muscle mass shall at least partly contribute to the loss of muscle strength which in turn contribute to the decline in muscle function due to the impaired ability to overcome resistance forces involved in the physical activity. In fact, the association between muscle mass and strength was supported by early cross-sectional studies until recent longitudinal studies demonstrated that there may be a disassociation between age-related changes in muscle mass (sarcopenia) and strength (dynapenia), and there are distinct mechanisms accounting for each component [15, 16]. These recent evidences not only bring a new terminology “dynapenia” into the field but also a reconsideration of the mechanism and etiology that underlies the problems as well as examining the problem from other perspectives. However, according to the original definition of sarcopenia [1],

*no decline with age is as dramatic or potentially more significant than the decline in lean body mass. In fact, there may be no single feature of age-related decline more striking than the decline in lean body mass in affecting ambulation, mobility, energy intake, overall nutrient intake and status, independence and breathing. Irwin H. Rosenberg, 1988*

Notwithstanding, the focus is on the decline of lean body mass, and if scrutinized carefully, the statement is actually referred to a phenomenon that relates to both the body mass and function but not the strength. Thus, “sarcopenia” in 1988 is defined as the change of body functions and behavior due to the loss of lean body mass. However, as strength is an independent predictor of body functions including falls and mortality [4, 16], it eventually replaces the body function and becomes part of the definition of sarcopenia in some studies [2, 3]. Since the definition of sarcopenia plays a central role in its research affecting the significance of its related research findings thereafter, we should have a consensus regarding this definition. Recently, some studies have pointed out the dissociation between the muscle mass and muscle strength [4, 15, 16] and have introduced the concept of “dynapenia” into the field; the strength parameter should be isolated from the definition of sarcopenia once again. However, when reading the original Albuquerque statement, it should be noted that Dr. Rosenberg has actually used the term “lean body mass” which includes the mass of muscles, blood, bones, and other nonfat tissues. All these tissues could experience aging and contribute to the decline in body function at a certain degree. Even the prefix “sarco” is commonly used to depict the muscles, and the Greek translation for “sarx” is only flesh whilst the one for muscle is “myx”. As a result, the term “myopenia” may be more appropriate for the current pathological condition of the age-related decline in muscle mass.

Virtually all diseases and pathological conditions involve a change in body physiology in which a phenotype is determined by a myriad of molecular factors that weave different molecular cascades in an interlacing fashion. Interestingly, one physiological factor may link up to several diseases (e.g., inflammation involved in both Alzheimer's disease and Parkinson's disease) and one sort of disease may also have several underlying physiological factors (e.g., amyotrophic lateral sclerosis has several subtypes and each of which is associated with a different gene or protein). In the case of sarcopenia, although muscle wasting and muscle atrophy are main features of its pathology, it is not unique at least Cachexia has a similar characteristics and consequence as sarcopenia [8, 17, 18]. The certain degree of pathological overlapping suggests that at least some underlying molecular mechanisms are common to/shared between these pathologies. For instance, inflammation where proinflammatory cytokines and ubiquitin-proteasome degradation pathway are upregulated [17]. Despite the similarity and overlapping characteristics with cachexia, sarcopenia is a separate clinical condition with a certain degree of discrepancies. Sarcopenia is a chronic muscle wasting condition associated with a low grade systemic inflammation which is not necessary to be the pathological trigger whilst cachexia is an acute muscle wasting condition that only develops under an overlying inflammation [8, 17]. In summary, it may be more appropriate to account the loss of muscle mass in subtypes or in a way similar to the definition of amyotrophic lateral sclerosis. For example, the subtype with a muscle-in-origin etiology shall call “type I sarcopenia” whilst the subtype with a neuron-in-origin etiology shall call “type II sarcopenia”. Or from the other perspective, the term “sarcopenia” shall be a pronoun for all types of chronic muscle mass decline and each of its subtypes is defined by one sort of etiological mechanism. For instance, the one shared with cachexia shall be called “cachexic sarcopenia” whilst the one shared with amyotrophic lateral sclerosis shall be called “Hawking's sarcopenia.”

## 3. Commonly Accepted Etiology of Sarcopenia

**Figure 1:**
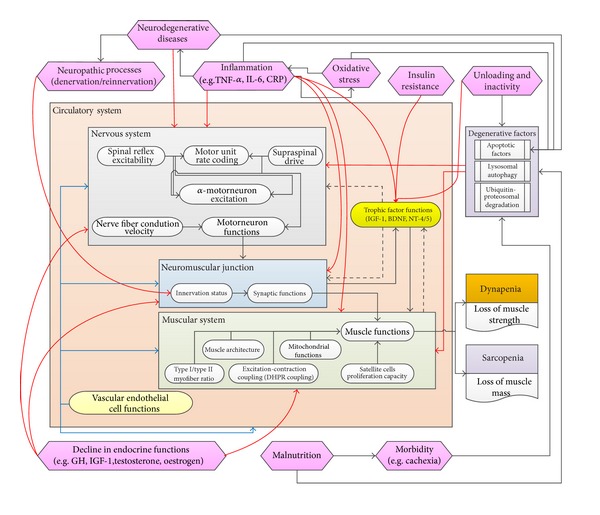
Summary of the neuromuscular mechanism and its relationship with the commonly accepted etiological factors of sarcopenia. The relationships between each of these factors are interlaced in a complex fashion. Black solid lines = positive regulators/reinforcements; black dash lines = positive regulators from the muscular system; red solid lines = negative regulators; blue solid lines = regulation by the circulation.

Currently, the body physiology or the etiology of sarcopenia is believed to be affected by but not restricted to five main features: (1) aging, (2) genetics, (3) morbidity, (4) nutrition, and (5) activity. Nevertheless, it is certain that muscle mass, strength, function, and quality are determined by at least three cardinal physiological systems: (1) neurological system [15], (2) muscular system [15], and (3) circulatory system. Thus, it is not surprising that any diseases or conditions that affect or alter these physiological systems may contribute to the development of dynapenia and sarcopenia, for example, genetic factors, neurodegenerative diseases, hormonal dysregulatory diseases, autoimmune diseases, inflammation, malnutrition, physical injuries, and inactivity [5, 8, 12]. As the muscle function relies on the status of motor units (each of which consisted of a motor neuron, motor neurites (axons), neuromuscular junctions (NMJ), and muscle fibers) in addition to the circulation factors, the pathology of sarcopenia may be neurogenic, musculogenic (term created to differ from “myogenic”), synaptogenic (from NMJ), and/or vasculogenic (from blood vessels) (Figure 1).

### 3.1. Muscular Factors

Aging is a symplcetic (“sym”: together; “plektos”: braid) natural process of matters and contributes significantly to sarcopenia. For skeletal muscle, aging leads to a decline in muscle function. At the systemic level, both the upper and lower limbs of men and women have an age-associated sequential loss in muscle power, muscle strength, and muscle mass, starting from the age of 40 years, 30 years, and 24 years, respectively [19]. The age-related loss of muscle power is more rapid than the parallel loss of muscle strength which in turn is more rapid than the loss of muscle mass [12, 19]. The loss in muscle power and muscle strength is at least partly attributable to the reduction in muscle mass. At the molecular level, the loss of muscle power and muscle strength is further associated with a reduction in the amount of Ca^2+^ available for the mechanical response in muscles [20] and with a reduction in Ca^2+^ release in response to the mechanical action of muscles due to the age-associated reduction in dihydropyridine receptors at the t-tubule and sarcoplasmic reticulum membrane which in turn results in uncoupling of Ca^2+^ release channels or ryanodine receptors in type II muscle fibers [15, 19, 20]. On the myocellular level, skeletal muscle fibers are basically two types. Type I fibers are slow and oxidative which mainly operate in the weight bearing/antigravity functions, whilst type II fibers are fast and glycolytic which are mainly involved in the explosive actions (e.g., sprinting). Patients with sarcopenia manifested a reduction in the number of both type I/slow-twitch and type II/fast-twitch muscle fibers and an atrophy specific to the type II fibers (being more prominent in IIB fibers than IIA fibers) [12, 19]. This may be resulted from a hypotrophic or hypercatabolic state of muscle fibers which may be a cause or an effect of the denervation of motor units and/or the loss of motor neurons [21]. These are the common features of aging and are being more prominent in type II than type I fibers. However, the hypercatabolic state of muscle fibers may also attribute to the reduction in the number of satellite cells [22]. In other words, the loss of muscle mass is also partly attribute to the diminished ability of muscle self-repair due to the decreased number and impaired function of satellite cells [2, 22]. At the cellular level, aging is associated with a reduction in cell density of the satellite cell population in type II fibers as well as a diminished satellite cell proliferation capacity or a replicative senescence, which may relate to the shortening of telomeres [2, 22, 23].

### 3.2. Neurological Factors

On the neurological side, the loss of muscle power and muscle strength is associated with the age-related changes in motor units and in the degree of coactivation of antagonist muscles, respectively [19]. At the cellular level, aging is associated with a reduction in motor axon conduction velocity and the number of myelinated axons. Further, it is associated with a reduction in motor unit reinnervation after denervation, and with a reduction in the number of motor units and motor neurons specific to the type II muscle fibers [19, 21]. As the fast-twitch motor units are the determinant of the degree of power exerted by muscles, their loss in aging contributes to the loss in muscle power [19]. In fact, under normal aging process, a preferential denervation of type II muscle fibers occurs and these denervated fibers are then reinnervated by the axonal sprouting from slow motor neurons in a process called motor unit remodeling. However, if denervation outpaces reinnervation, then a population of denervated fibers will undergo atrophy and degeneration [24] due to the loss of trophic factors [25]. This process contributes to the loss of muscle mass at least partly by apoptosis [26]. At the molecular level, rat studies have shown that progressive denervation during aging have disrupted the precise overlapping between the presynaptic nerve terminal and the postsynaptic acetylcholine receptor (AChR) clusters at the NMJ [27]. Also, denervated muscles have elevated the expression of proapoptotic/atrophic factors including bax, caspase 3, 7, 8, and 10 [26] along with a reduction in the trophic factor signals including TrkB signaling via BDNF and NT-4/5 [27]. These in turn increase the apoptotic potential of myocytes. Thus, denervation seems to be the trigger for muscle loss.

### 3.3. Circulation Factors

Blood circulation is vital to the whole body and thus may also contribute to the pathology. Aging is associated with the changes in microcirculation and ultrastructure of the vascular endothelium [28], a decline in vascular endothelial functions [29], and a decline in exercise-induced blood flow which may be partly resulted from an age-related reduction in vasodilatory capacity and capillarization [28]. The reduced blood flow leads to a reduction in the exchange of oxygen, energy sources, metabolites, and heat between blood and the body cells. This results in a less trophic environment for the cells in the corresponding region [28].

## 4. Common Pathophysiology Shared between the Aging Nervous System and the Aging Muscular System

According to most of the studies, contributors to the loss of muscle mass in sarcopenia are explained mainly by, but not limited to: (1) mitochondrial dysfunction, (2) elevation of oxidative stress, (3) inflamm-aging, (4) altered rate of protein turnover, (5) decreased hormones, growth factors, and proteins that maintain proper cellular functions, (6) declines in intake of essential nutrients, and (7) declines in physical activity [5, 12, 19, 21]. Interestingly, most of these contributors are not unique to the muscular system. They may also applicable to the nervous system.

### 4.1. Mitochondrial Dysfunction

Mitochondrial dysfunction may arise from the age-related decline in mitochondriogenesis [14, 30] and mitochondrial function. Both of which may be associated with an increase in the mitochondrial DNA damage by oxidative stress, changes in the mitochondrial respiratory chain enzymes, and changes in the mitochondrial proapoptotic proteins. Mitochondrial dysfunction results in an impaired mitochondrial oxidative function and energy production. These in turn affect cell viability through necrosis and/or apoptosis of both myocytes and neurons [21, 31].

### 4.2. Elevation of Oxidative Stress

Elevation of oxidative stress is commonly seen in aged animal cells with an increase in the oxidative species (e.g., H_2_O_2_ species, MDA/4-HAE, nitrotyrosine, catalase [32], iNOS [17]) and a decrease in the antioxidative species (e.g., MnSOD [32], G6PDH [33]). Also, accumulation of the oxidative stress-induced advanced age glycation end-product (AGE) and lipoxidation end-products (ALE) on the extracellular and intracellular proteins during aging impairs the activities of these proteins [21]. The consequence of an elevated oxidative stress in muscles is the iNOS-mediated downregulation of a set of myogenic proteins including CKM and MyoD [17], and the concomitant inhibition of general protein translation by both the phosphorylation of eIF2*α* and the inhibition of mTOR [17]. Additionally, aging upregulates the iNOS which correlates with an increase in caspase 2 and JNK signaling activity, this suggests the involvement of JNK-mediated apoptotic signaling [33]. In the perspective of the nervous system, oxidative stress may lead to an alteration in the balance between mitochondrial fission and fusion as well as an activation of the apoptotic pathway in neurons [34].

### 4.3. Inflamm-Aging (Aging-Associated Chronic Inflammation)

Inflammation is intimately linked to the oxidative stress. Inflamm-aging is an age-related elevation of the baseline level of proinflammatory markers and cytokines (e.g., TNF-*α*, IL-6, CRP) [10]. The most representative cytokines are TNF-*α* and IL-6. TNF-*α* induce apoptosis directly through its iteraction with the death domain receptors which in turn leads to the activation of procaspase 8 [21, 26]; and indirectly through the activation of its downstream effectors, NF-*κ*B, which activity is also elevated by aging [17]. Activated NF-*κ*B upregulates MSTN/GDF8 [35], iNOS, and MuRF1 [17], these factors play a negative role in the trophic state of myocytes. Additionally, IL-6 plays a similar role by downregulating IGF-1 [2]. As a result, the anabolic potential of myocytes including the satellite cells is impaired due to (1) reduced expression of proteins involved in myogenesis and other relevant muscle growth processes (e.g., MRF, myogenin, MyoD, and CKM) [17, 26]; (2) elevated expression of MSTN which exerts its effect on muscle growth by regulating the Activin receptor-mediated pathway, MAPK pathway, and Akt/TORC1/p70S6K pathway [5]. Additionally, MSTN induces reactive oxygen species (ROS) production via NADPH oxidase and TNF-*α*. The elevated TNF-*α* in turn induces further MSTN production and the higher levels of MSTN promotes proteasome-mediated catabolism of intracellular proteins [35]. In addition to the muscular system, both oxidative stress and inflammation have great impacts on the nervous system as they are commonly disclosed in the neurodegenerative diseases [34, 36].

### 4.4. Altered Rate of Protein Turnover

Altered protein balance is one of the major features in aging cells. In myocytes, there is an age-associated change in expression of dystrophic factors (e.g. Id1, Id2 and Id3, etc.) [26] and trophic factors (e.g. MGF, etc.) [19]. Similarly, the expression profile of dystrophic/proapoptotic factors (e.g. bax and procaspase 3, etc.) [37] and trophic factors (e.g. CNTF, etc.) [19] in neurons are also altered by aging. The fate of a cell is determined by the balance between the rate of positive metabolism and the rate of negative metabolism which are positively correlated to the level of trophic signal (e.g., growth factor-mediated pathways) and atrophic/dystrophic signal (e.g., proapoptotic factor-mediated pathways), respectively. Thus, when the rate of negative metabolism exceeds the rate of positive metabolism, the cell will undergo atrophy and finally death by apoptosis. In the case of aging myocytes, despite the mechanism is not clear, the loss of multinucleated myocyte nuclei is suggested to be caused by apoptosis regulated by AIF-mediated caspase-independent DNA fragmentation. Unlike single-nucleated neurons, apoptosis of one single nucleus of the myocyte may not result in cell death [21]. Instead it will undergo an atrophy due to the decrease of the nuclear domain [19]. Together with the “use it or lose it” perspective (discuss in later section), this may explain the more remarkable reduction in the size of type II muscle fibers than that of the type I muscle fibers in sarcopenia. Additionally, this also suggested that myocytes may have a greater potential to resist death than neurons. At the transcription level, miRNAs may also contribute to the age-related alteration in protein turnover as they are capable of regulating protein expression. Many miRNA candidates altered their expression level during aging in both myocytes and neurons [38–40].

### 4.5. Decreased Hormones, Growth Factors, and Functional Proteins

Hormones, growth factors, and proteins that maintain proper cellular functions are associated with the trophic state of myocytes. Aging is associated with a decline in sex hormones in both male and female, for example, androgen and estrogen [2]. On the neurological side, the alteration in sex hormone levels may also affect brain functions as circulating sex hormones can penetrate the blood-brain barrier [41]. Additionally, aging is associated with a decline in growth factors and their relevant regulators affecting both myocytes and neurons, for example, (1) GH, which regulates the synthesis of IGF-1 [19] and the survival of neurons [42]; (2) IGFs, which stimulate amino acid and glucose transport and are important trophic factors to both myocytes [5, 12] and neurons [43–45]. IGF-1 regulates growth, differentiation, and regeneration of myocytes [12] by inducing hypertrophy pathways (e.g., PI3K and MAPK pathways) [5] whilst IGF-2 is associated with the proliferative action in adult muscles [12]; (3) CNTFs, which are important hypertrophic factors for both myocytes and neurons [12] and may play a role in the re-innervation of muscle fibers by motor neurons after muscle and nerve injury [5]. Apart from the growth factors, aging is also associated with a reduction in stress-induced expression of HSP70, which normally functioned as chaperones and expressed by both myocytes and neurons [21, 23]. HSP70 reduces the apoptotic potential of a cell by inhibiting the formation of apoptosome and functioning as an antagonist of AIF [21]. Thus, the age-associated decline in these candidates may have a serious consequence on both the muscular and nervous systems.

### 4.6. Malnutrition

Aging is commonly associated with a decline in the ability to utilize exogenous amino acids. This may be due but not limited to: (1) reductions in transmembrane amino acid transport for protein synthesis; (2) alterations in the whole body amino acid turnover which results in a reduced availability of substrate for protein synthesis; (3) alterations in the endogenous hormonal response; and/or (4) alterations in the response of muscle to the hormonal stimuli after meal intake [47]. The current recommended daily nutrition intake for the prevention of sarcopenia and frailty is 24–36 kcal and 0.8–1.2 g high quality protein per kg body weight [9]. Essential amino acids, in particular the branched-chain amino acids (BCAA) especially Leucine, are potent anabolic signals for protein accretion [47, 48], which requires ~0.7 kcal/g of muscle protein synthesized [47]. Therefore, lacking such nutrients may alter the protein turnover and thus contribute to the development of sarcopenia [8].

### 4.7. Disuse Atrophy

Physical inactivity, induced by either sedentary lifestyle or immobility due to illness/injury, is a trigger of muscle disuse atrophy [18]. At the molecular level, physical inactivity is associated with an inhibition of the IGF-1 hypertrophic signaling and a concomitant upregulation of proteasomal (ubiquitin) and lysosomal (autophagy) degradation signaling via FOXO proteins [18, 49]. This contributes to a reduced trophic state in myocytes. In contrast, increased physical activity level by resistance training enhances the muscle mass, muscle strength, and balance which in turn reduces the risk of physical limitation and/or the onset of frailty [11, 21]. The effect of exercise training is dose dependent. The higher the intensity involved in the training, the better the yield of the effect. Training at 60%–85% of the individual maximum voluntary strength can increase the muscle mass whilst more than 85% can also increase the rate of force development. The addition of a sensorimotor component to the exercise training program may also help improving the postural control in older persons [50]. In addition to the muscles, the influence of exercise training to the nervous system is also well described [51]. Interestingly, although having a positive impact on both muscle mass and strength, exercise trainings do not prevent the age-related impairment in muscle features. Recent studies reported that both the muscle size and functions are impaired in aged sprint-trained athletes. Similar to sarcopenia, type II muscle fibers were suffered from a more remarkable impairment than type I muscle fibers [52].

## 5. Use It or Loss It

On the neurological perspective, physical inactivity reflects a reduced activity of the corresponding motor units. Unused or seldom used neurons will undergo disuse degeneration which in turn results in a further disuse degeneration of its synaptically connected cells [53]. Thus, it is possible that the age-associated progressive denervation and the age-associated loss of muscle fibers are due to the disuse atrophy and degeneration of the NMJ and/or the motor neurons. This “use it or loss it” doctrine at least partly explains why there are more prominent type II motor unit atrophy and degeneration in sarcopenia and why sedentary individuals are more susceptible to sarcopenia. As sedentary individuals have less explosive actions, the frequency of usage and/or length of activation of the fast motor unit (type II fibers) is lesser than that of the slow motor unit (type I fibers) which is frequently engaged in the antigravity functions. Thus, the potential of disuse atrophy and degeneration for type II fibers is higher. In fact, age-related denervation, loss of motor units, and motor neurons are all being more prominent in the type II fibers in comparison to the type I fibers [19, 54]. Further, space flight studies reported that the loss of muscle mass, strength, and power are more prominent in the type I fibers than those of the type II fibers under microgravity [55]. These may be explained by the “use it or loss it” doctrine again. As gravity is reduced, the usage of slow motor unit is lesser than that of the fast motor unit. Hence the potential of disuse atrophy and degeneration for the type I fibers is higher. It is interesting to note that after the space flight, the type II fibers were also lesser. This may due to the reduction in gravity which leads to the reduction in resistance force for activity, and the type II motor units were then using lesser strength, energy, or power for the same work. As a result, they were susceptible to the disuse issue but to a lesser degree.

## 6. The Origin of Sarcopenia

Contributors to the development of sarcopenia discussed so far are common to both myocytes and neurons. Thus, the aforementioned contributors may be the systemic/general factors which may not necessary be the primary trigger in the etiology of sarcopenia but rather they may play a role in exacerbating the development process of sarcopenia upon triggering. Apparently, sarcopenia is linked to the interplay of assorted molecular cascades which involve a complex relationship between a variety of nucleic acids and proteins. Therefore, it is also possible that the pathology is originated from these multiple independent or synergistic triggers. Additionally, morbidity factors including neurodegenerative diseases and other pathological conditions may also have a direct or indirect role in the development of sarcopenia, that is, sarcopenia as a complication of other diseases.

**Figure 2:**
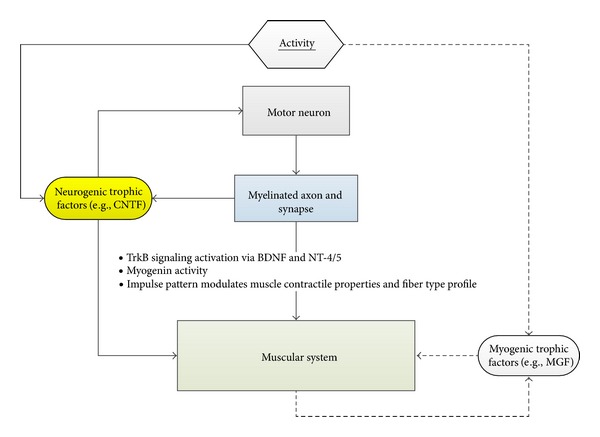
Relationship between the nervous system and the muscular system. (1) CNTF is released from the peripheral nerves and is one of the vital trophic factors for both the motor neuron and the myocytes. This growth factor is abundantly synthesized by Schwann cells. (2) MGF is locally released from the muscles and mainly activates satellite cells proliferation. (3) Some trophic factors (TrkB and myogenin) are innervation dependent. (4) Electrical impulse pattern modulates the muscle contractile properties and fiber-type profile.

By putting these pictures together, the development of sarcopenia may originate either from the following: (1) muscle fiber atrophy and degeneration which in turn leads to the denervation and loss of motor neuron (musculogenic); (2) synapse atrophy and degeneration of the NMJ which in turn leads to the atrophy and degeneration of muscle fibers (synaptogenic); (3) motor neuron atrophy and degeneration which in turn leads to the atrophy and degeneration of muscle fibers (neurogenic); (4) all of these. On the other hand, the exercise-induced hypertrophy of muscles may be explained either by (1) hypertrophy of the muscle fiber which in turn reinforces and stabilizes the synapses (muscle-induced); (2) hypertrophy of the synapses at the NMJ by synaptic facilitation through reinforcing signals and this in turn potentiates the trophic status of the muscle fibers (nerve induced); (3) both. Interestingly, the nervous system-based ideas seem more feasible due to (Figure 2) the following: (1) the trophic state of the muscle is determined by the net protein balance and some myogenic factors, e.g., myogenin, are strongly regulated by the electrical activity [25]; (2) some trophic factors, e.g., NT-4/5 and TrkB, are innervation dependent [56]; (3) some neurotrophic factors, CNTF, nourishes both the muscular and nervous system [57]; (4) the loss of muscle mass and strength albeit having different onset time, both of them are well maintained or decline slowly until the age of 60 when a more rapid rate of decline commences. Coincidentally, the onset of age-associated denervation is also around the age of 60 [19]; (5) physiological change in the presynaptic terminal is greater than the postsynaptic terminal in aged animals [27]; (6) the nerve activity determines the muscle phenotype and this is mediated mainly by the Ras-MAPK pathway [25]; (7) the neurotrypsin-dependent NMJ degeneration results in a full sarcopenia phenotype in young adult mice [58].

## 7. One Picture Has Two Sides

Nature is amazing and sometimes surprising, this makes science even challenging and fascinating especially when analyzing a particular phenomenon with a different or even antagonistic logic. For example, although hypertrophic state is commonly considered to be important for the survival of cells, chronic hyperactivity of growth-promoting pathways may not possess any positive impacts. Animal studies reported that hyperactivation of the mTOR pathway, which originally promotes accretion and regulates initiation of translation, does not reverse the atrophy observed in obese muscles [59]. In contrast, when treated with the AMPK-agonists which inhibit the mTOR pathway, the translation capacity and the mass of obese muscle increased [59]. Thus, hyperactivity of mTOR may lead to a secondary resistance to the growth stimuli probably due to the negative feedback of a homeostatic effect [17]. Interestingly, some human studies reported that the muscle mass is higher in obese old persons than those with lesser obesity [60, 61]. This may be due to the decreased levels of SHBG which in turn results in an increased level of circulating androgens [61].

When we considered food intake as an important factor for the prevention of sarcopenia, caloric restriction without being malnutrition may have a positive impact on cells [59]. Animal studies shown that caloric restriction attenuates the progressive functional decline of organs [21]. At the cellular level, caloric restriction is associated with a decreased damage of peripheral nerve during aging by increasing the expression of chaperones and autophagic machineries [24]. It also ameliorates the loss of muscle mass, decreases abnormalities in the electron transport chain and diminished apoptotic potential in muscles [21]. Further, it is associated with neuronal protection against degeneration in animal CNS [62]. However, more ongoing investigations in humans are required to determine its efficacy in reality.

Every picture has two sides, like the above examples, although the nervous system may have an important vital role for the muscular system, the muscular system is also playing an important vital role for the nervous system as the muscle fibers are supplying the NGF-like factors to the motor neuron for its survival [63]. Thus, when considering the motor unit holistically, the common musculogenic model of sarcopenia is also valid.

## 8. Conclusion

Sarcopenia is an age-associated condition which links to multiple etiological factors ranging from external factors (physical activities, nutrients, and diseases) to internal factors (interplay between different cells, epigenetic profile of the cells, and congenital genetic configuration of an individual). In this article, we propose that sarcopenia may be neurogenic in origin due to the intimate relationship between the nervous system and the muscle cells. By summarizing the aforementioned evidences, it seems that the pathological importance at the presynaptic side (nerve side) precedes the postsynaptic side (muscle side). This presumption is in agreement with the age-associated degeneration of motor neurons [21]. Further, the age-associated denervation is more likely to be triggered by the disuse atrophy of synapse or even motor neurons. This is consistent with the fact that individuals with a sedentary life style or physical inactivity are usually more susceptible to sarcopenia compared to the physical active ones. However, more investigation is required in order to validate this belief. For example, to perform an in vitro experiment on the primary muscle cells which cultured with the necessary nervous-system-derived factors and observe whether the change in these factors would significantly affect the potential for sarcopenia development. But before that, a consensus for the definition of sarcopenia must be committed.
